# Innovation in Aluminum Dross Processing: Comprehensive Strategies for Safe Disposal and Efficient Utilization in China

**DOI:** 10.3390/ma19153179

**Published:** 2026-07-25

**Authors:** Ruiying Wang, Fei Gao, Delei Chen, Zhenxing Yin, Minghui Li, Kang Mao, Canhua Li

**Affiliations:** 1 School of Metallurgical Engineering, Anhui University of Technology, Ma’anshan 243002, China; 2 MCC Huatian Engineering and Technology Corporation, Ma’anshan 243002, China; 3 State Key Laboratory of Environmental Geochemistry, Institute of Geochemistry, Chinese Academy of Sciences, Guiyang 550081, China

**Keywords:** primary aluminum dross, secondary aluminum dross, harmless treatment, resource utilization

## Abstract

With the rapid development of the global aluminum industry, the production of aluminum dross, as a hazardous solid waste generated in the aluminum production process, has increased annually. Aluminum dross contains not only substantial amounts of harmful substances, such as aluminum nitride, fluoride, and chloride, but also valuable resources such as metallic aluminum and alumina. This review summarizes the sources, composition, and hazards of aluminum dross to the environment and human health, with a focus on the harmless treatment and resource utilization technologies for aluminum dross. Harmless treatment technologies include denitrification, defluorination, and dechlorination, which are primarily classified into hydrometallurgical and pyrometallurgical methods. In terms of resource utilization, primary aluminum dross (PAD) was mainly used to extract metallic aluminum through heat treatment and cold treatment, while secondary aluminum dross (SAD) can be used to prepare refractory materials, building materials, water treatment agents, and extract alumina. This review also compares the advantages and disadvantages of current aluminum dross treatment technology and outlines future research directions, aiming to provide a reference for the sustainable development and environmental protection of the aluminum industry.

## 1. Introduction

**Figure 1:**
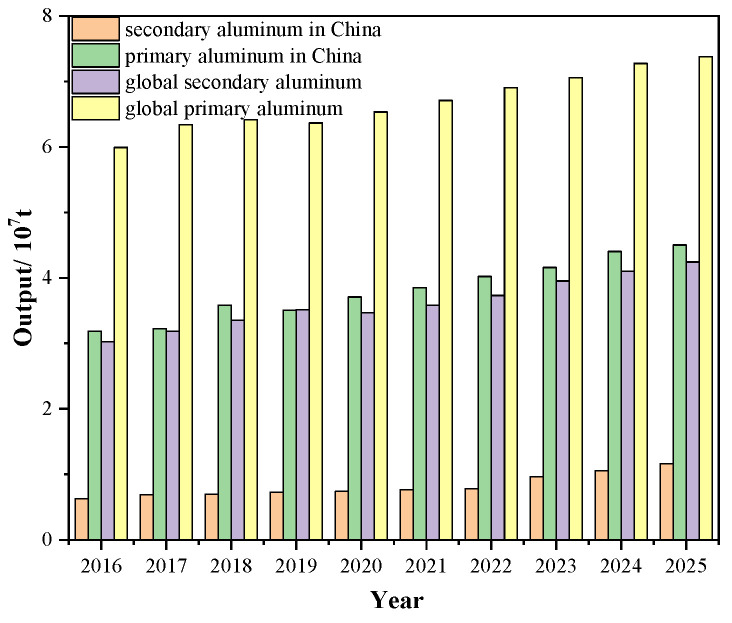
Production trend of PAD and SAD in the world over the last ten years.

Aluminum dross is a hazardous solid waste generated during aluminum production. Aluminum dross is classified internationally into white dross and black dross, while PAD and SAD are the conventional terms adopted in the Chinese academic literature. With the rapid development of the global aluminum industry, the production of aluminum dross has increased annually. Figure 1 illustrates the production trends of PAD and SAD production in the world in the past decade. As shown in Figure 1, global primary aluminum production increased steadily between 2015 and 2025 [1,2]. Global secondary aluminum production exhibited a similar steady upward trend over the same period [3,4]. In 2025, China’s primary aluminum production reached 45.03 million tons, and the production of secondary aluminum was about 11.60 million tons, accounting for 61.03% and 27.36% of the global totals, respectively. In the production process of the aluminum industry, the primary and secondary aluminum processes generate 3–5% of PAD and 8–15% of SAD per 1t of molten aluminum [5]. Based on these data, China’s current annual aluminum dross generation has exceeded 3 million tons.

In 2020, aluminum dross was officially included in China’s National Hazardous Waste Catalogue, belonging to the waste non-ferrous metal mining and smelting (HW48). The annual production of aluminum dross in China was as high as more than 3.5 million, and most of the aluminum dross was treated by landfill, which poses a great risk to the environment [6]. The stacking and landfill treatment of aluminum dross not only occupies a large amount of land resources but also causes pollution to the soil and groundwater. In a humid environment, the aluminum nitride in aluminum dross would react with water to generate ammonia, resulting in air pollution and direct harm to human health [7]. On the one hand, the landfill treatment of aluminum dross can cause serious harm to the environment and human health; on the other hand, aluminum dross is not a simple waste product, it is a resource rich in valuable materials such as metallic aluminum and alumina, showing great development potential and application prospects in the field of resource recycling, and it can also yield significant economic and environmental benefits. Therefore, how to realize the resource utilization and harmless treatment of aluminum dross not only concerns the sustainable development of the aluminum industry but is also of great significance for environmental protection. In response to the national concept of green and low-carbon processes, exploring efficient and environmentally friendly aluminum dross treatment and resource utilization technology has become the key to solving the problems affecting the sustainable development of the aluminum industry.

A systematic literature search was conducted covering publications from 2008 to 2025. The search was performed in CNKI, Google Scholar, and Web of Science using keyword combinations including ‘aluminum dross,’ ‘primary aluminum dross,’ ‘secondary aluminum dross,’ ‘harmless treatment,’ ‘denitrification,’ ‘defluorination,’ ‘dechlorination,’ ‘resource utilization,’ ‘refractory,’ ‘building materials,’ and ‘alumina extraction.’ Relevant peer-reviewed studies were systematically evaluated and categorized according to the thematic structure of this review.

### 1.1. Source and Chemical Composition of Aluminum Dross

Aluminum dross originates primarily from processes such as electrolytic aluminum, casting aluminum and recycled aluminum. During electrolytic aluminum processing, refractory impurities and additives that are difficult to melt accumulate on the surface of molten aluminum and oxidize upon contact with air, thereby forming dross. Additionally, the replacement of the cathode lining and equipment maintenance in the electrolytic cell will also produce solid waste. During aluminum casting operations, the alloying treatment of electrolytic aluminum liquid and casting will produce a large amount of aluminum dross. As primary aluminum resources become increasingly scarce, the recycled aluminum industry has gradually shifted to using scrap aluminum and aluminum ingots as the main raw materials. During the processing of these materials—covering remelting, refining, alloying, and molten casting—aluminum dross is generated at multiple stages, from surface oxidation of scrap aluminum to impurity precipitation during refining, reaction products during alloying, and residual surface materials during casting. Each ton of molten aluminum can produce 80–150 kg of aluminum dross residue, which is discarded as solid waste because its quality does not meet the requirements of the aluminum smelting industry [8].

**Figure 2:**
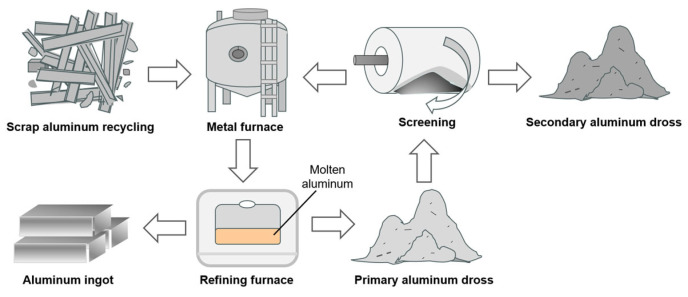
Flow chart of the process for the production of recycled SAD.

Based on the production process and aluminum content, aluminum dross is generally divided into PAD and SAD. PAD was a by-product directly generated in the process of electrolytic and casting aluminum processing, which is rich in metallic aluminum, with a gray or white granular appearance, and it could be directly recycled as secondary aluminum industry raw materials [7]. SAD, as shown in Figure 2, was the waste produced after the remelting or recycling of aluminum ingots from PAD, with lower metallic aluminum contents and higher salt and harmful substance content, appearing as fine black particles and being more difficult to process [6,9,10].

The actual compositions of both PAD and SAD vary significantly depending on the production route, raw materials, and subsequent treatment stages. Table 1 summarizes the typical compositional ranges and their primary sources of each type of aluminum dross.

The chemical composition of SAD is shown in Table 2, the chemical composition of SAD varies considerably across different sources.

**Figure 3:**
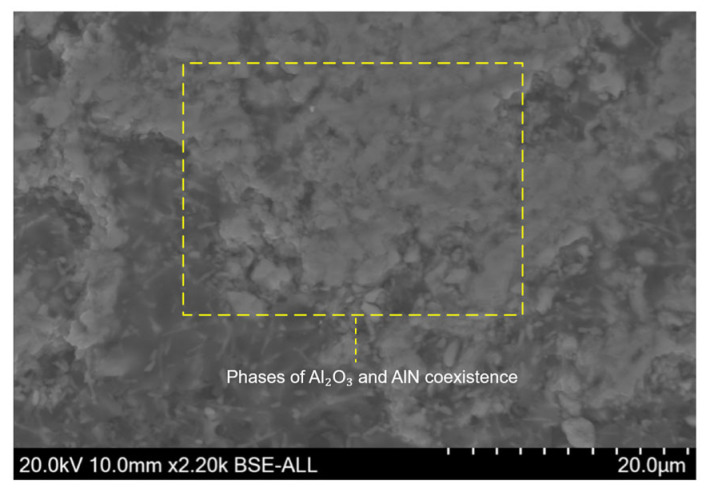
SEM image of SAD [14].

The mineral phases of SAD are as important as its chemical composition, as they determine both the environmental hazards and the potential for resource utilization. SAD contains substantial amounts of alumina (Al_2_O_3_), magnesium aluminate spinel (MgAl_2_O_4_), and aluminum nitride (AlN) [13]. MgAl_2_O_4_ spinel offers excellent thermal shock resistance and corrosion resistance, making it a superior raw material for high-value refractory applications. The coexistence of these phases—hazardous AlN requiring removal, recoverable Al_2_O_3_, and value-added MgAl_2_O_4_ spinel—highlights the dual nature of SAD as both an environmental liability and a potential resource. The average particle size of SAD ranges from approximately 4 to 20 μm, and is largely dependent on the pretreatment conditions [14,15]. This fine particle size is favorable for enhancing the leaching efficiency and sintering reactivity of SAD. SAD powder, depicted in Figure 3, exhibits well-defined regular shapes and smooth surfaces, with almost no adhered fine particles.

### 1.2. The Hazards of Aluminum Dross

Given the significant challenges that aluminum dross poses to human health and the environment, the Ministry of Ecology and Environment of People’s Republic of China issued the “National Hazardous Waste Catalogue (2025 Edition)”, which included PAD generated on the surface of molten metal during the electrolysis of aluminum liquid, as well as SAD generated during the recycling of aluminum [16]. Since the implementation of the “Environmental Protection Tax Law of the People’s Republic of China”, the disposal of hazardous solid waste such as aluminum dross is subject to an environmental protection tax at 1000 CNY per ton [17]. Under these policy constraints, the environmental protection supervision faced by aluminum dross-producing enterprises has become increasingly stringent, and the cost of compliance disposal has increased significantly. How to achieve the safe disposal of aluminum dross and its high-value-added resource utilization has become a key issue for industrial development. Aluminum dross was classified as hazardous waste due to its complex chemical composition and high content of toxic substances, which posed significant environmental hazards. It must accord to hazardous waste management protocols and not be discarded arbitrarily [8]. Aluminum dross contains aluminum nitride, aluminum carbide and metallic aluminum, which have strong chemical activity and would rapidly undergo hydrolysis reaction upon contact with water, releasing harmful gases such as ammonia, methane, and hydrogen [9]. Ammonia is toxic and irritating, while methane and hydrogen are not only flammable and explosive but may also cause explosions under certain concentration conditions, posing serious safety hazards and environmental risks during storage, transportation and disposal. The equations for the hydrolysis of AlN, Al_4_C_3_, and Al are shown in Equations (1)–(3). Notably, the reaction in Equation (3) is kinetically hindered at room temperature due to the native Al_2_O_3_ passivation layer, resulting in negligible H_2_ evolution. This reaction becomes significant only under harsh thermal conditions or in the presence of aggressive ionic species (e.g., Cl^−^ salts).

AlN + 3H_2_O = Al(OH)_3_ + NH_3_, (1)

Al_4_C_3_ + 12H_2_O = 4Al(OH)_3_ + 3CH_4_, (2)

2Al + 6H_2_O = 2Al(OH)_3_ + 3H_2_, (3)

Therefore, stockpiled aluminum dross poses a great safety hazard, and it may pose a serious threat to the environment and human health under rain or humid weather [9,10]. Moreover, the fluoride and chloride in aluminum dross have high water solubility, and random stockpiling will lead to the seepage of these harmful substances into the soil and groundwater, resulting in salinization and contamination of land and groundwater [6]. In addition, heavy metal ions such as As, Pb, and Cd in aluminum dross can also contaminate soil and water bodies, affecting crop growth and human health, and stockpiling also consumes substantial land resources, which is a waste [10].

In summary, from the production pathway and chemical composition analysis of aluminum dross, the temporary storage of aluminum dross causes a certain damage to the environment and ecology, and it is necessary to carry out harmless treatment so that aluminum dross can be used as a secondary resource.

## 2. Harmless Treatment of Aluminum Dross

**Figure 4:**
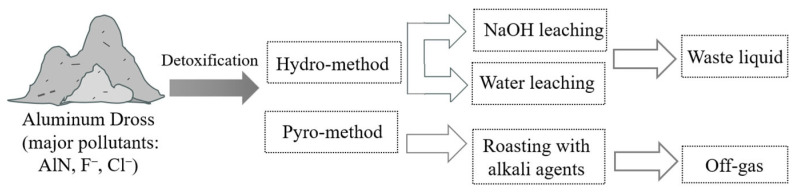
Main technological pathways for harmless treatment of aluminum dross.

Aluminum dross contains a large amount of harmful substances such as aluminum nitride, fluoride and chloride. Therefore, rendering it harmless is particularly important. Current detoxification technologies are primarily classified into hydrometallurgical and pyrometallurgical routes, targeting the removal or stabilization of these harmful components. Currently, hydrometallurgical and pyrometallurgical techniques each have their own advantages and disadvantages in denitrification, defluorination, and dechlorination. Hydrometallurgical technology offers the advantage of convenient operation; however, its treatment efficiency is limited to a certain extent. Pyrometallurgical technology can achieve efficient processing, but it consumes high amounts of energy during operation. Future research could attempt to develop combined hydro- and pyrometallurgical treatment processes, for example, using a hydro-method for pretreatment to initially remove harmful components such as nitrogen, fluorine, and chlorine, and then applying a pyro-method for deep treatment to achieve an optimal balance between treatment efficiency and energy consumption. Currently, traditional additives such as NaOH and CaO can effectively promote the reaction process and improve the treatment efficiency, but there are potential risks in practical application. The use of these additives may lead to secondary pollution, while also increasing production cost. Therefore, future research should focus on developing green and environmentally friendly additives, ensuring efficient pollutant treatment while minimizing negative impacts on the ecological environment. In addition, by-products such as ammonia and fluorine-containing wastewater generated during the harmless treatment process have high recovery value. With appropriate resource recovery technologies, these by-products can be converted into economically valuable products, which can not only reduce treatment costs, but also improve economic benefits and overall resource utilization efficiency. The mainstream technological pathways for aluminum dross detoxification are visually summarized in Figure 4 for intuitive understanding.

### 2.1. Denitrification Treatment

The removal of AlN is critical because its hydrolysis generates toxic and flammable NH_3_ gas. Denitrification is achieved through either hydrometallurgical hydrolysis or pyrometallurgical oxidative sintering. The denitrification performance of typical hydro-method and pyro-method processes is compared in Table 3.

#### 2.1.1. Hydro-Method

The hydro-denitrification method has some key problems, among which the encapsulation of hydrolysis products on aluminum nitride particles significantly inhibits the progress of the hydrolysis reaction, resulting in a gradual decrease in the reaction rate [21]. To effectively improve hydrolysis efficiency, reaction conditions can be optimized through several means: the precise control of hydrolysis temperature, reasonable adjustment of reaction time, and optimization of key parameters such as the solid-to-liquid ratio. These measures can effectively improve the reaction kinetics, thus significantly improving overall hydrolysis efficiency. Within a certain range, higher hydrolysis temperatures, longer reaction times, and better denitrification effects are correlated. Qi et al. [27] found that as the liquid-to-solid ratio increased, the nitrogen removal rate of SAD showed a slowly increasing trend. In the absence of additives, the hydrolysis products of aluminum dross are mainly Al(OH)_3_ and NH_3_. Using pure water to hydrolyze aluminum nitride results in aluminum hydroxide wrapping the aluminum nitride particles, which not only results in a low denitrification rate but also consumes large amounts of water. To improve the denitrification rate, additives can be added to optimize the hydrolysis process [28]. The hydrolysis reaction equations of AlN with NaOH are shown in Equations (4) and (5):

AlN + 3NaOH = Na_3_AlO_3_ + NH_3_, (4)

AlN + NaOH + H_2_O = NaAlO_2_ + NH_3_, (5)

During the hydrolysis of AlN, not only is Al(OH)_3_ produced, which hinders further hydrolysis, but soluble Na_3_AlO_3_ and NaAlO_2_ are also formed, covering part of the AlN surface and thus improving the denitrification rate. Lin et al. [29] proposed a calcium-driven hydrolysis method for denitrification: adding CaCl_2_ promotes the conversion of Al(OH)_3_ to Al(OH)_4_^−^, driving the hydrolysis of AlN, reducing the activation energy of the reaction, and thus improving the denitrogen rate. For the flammable ammonia gas produced during hydrolysis, freezing water can be used to recover ammonia as industrial ammonia water, which not only reduces pollution but also generates economic benefits [30].

#### 2.1.2. Pyro-Method

Oxidative sintering can eliminate the reactivity of aluminum dross, converting aluminum nitride into alumina and nitrogen. The sintering temperature has a significant effect on the denitrification rate of aluminum dross. When the temperature is between 50 and 900 °C, the denitrification efficiency of aluminum nitride increases significantly with temperature [6]. However, when the temperature rises to between 1000 and 1100 °C, the promoting effect of further temperature increases on the denitrification rate is significantly weakened. Therefore, to achieve the dual goals of efficient denitrification and cost control, in practical industrial applications, it is necessary to comprehensively consider the balance between the denitrification effect and the increased energy consumption brought about by increasing the temperature, as well as identify the optimal process parameters.

### 2.2. Defluorination Treatment

Fluorides are hazardous due to their high water solubility, which causes the contamination of soil and groundwater upon stockpiling. Defluorination is achieved by either hydrometallurgical leaching or pyrometallurgical high-temperature solidification. The defluorination performance of typical hydro-method and pyro-method processes is compared in Table 4.

#### 2.2.1. Hydro-Method

Hydrometallurgical defluorination removes soluble fluorides via water washing or alkaline leaching. Simple water washing is generally inefficient [32], but the addition of NaOH enhances fluoride dissolution by forming soluble fluoro-complexes or through competitive anion effects [19,31]. Fluoride ions can also be removed by adding calcium salts (e.g., CaCl_2_) to the wash solution, which react with fluorine ions to form CaF_2_ precipitates that are insoluble in water, thus achieving defluorination, but the use of calcium salts does increase treatment costs.

#### 2.2.2. Pyro-Method

Pyro-defluorination technology aims to convert fluorides in aluminum dross into stable compounds via high-temperature roasting, thereby achieving defluorination. By mixing aluminum dross with CaO and NaOH and roasting at high temperature, the fluorides are converted into stable CaF_2_, thereby achieving defluorination [33]. Although this approach achieves excellent fluoride stabilization (Table 4), it suffers from high energy consumption and the potential release of gaseous fluorides, necessitating stringent off-gas treatment systems.

### 2.3. Dechlorination Treatment

Chlorides originate from cover fluxes and refining agents used in aluminum melting. They are highly water-soluble and corrosive, requiring effective removal prior to disposal or reuse. Table 5 summarizes the relevant processes and dechlorination efficiencies of the hydro-method and pyro-method.

#### 2.3.1. Hydro-Method

Hydrometallurgical dechlorination is the most straightforward and cost-effective approach, as most chlorides are readily soluble in water. Water washing efficiently transfers Cl^−^ into the liquid phase, achieving removal rates exceeding 95% under optimized conditions [21,22,34]. Alkaline solutions can react with chlorides in dross to generate chlorides that are more readily soluble in water. Therefore, to improve the dechlorination rate, alkali can also be added to the aqueous solution [35]. The primary challenge is the generation of high-salinity wastewater, which can be managed through evaporative crystallization to recover NaCl and KCl as valuable by-products. The representative process data are summarized in Table 5.

#### 2.3.2. Pyro-Method

Pyrometallurgical dechlorination involves high-temperature roasting (typically >1000 °C) with additives (CaO, Na_2_CO_3_, NaOH), converting chlorides into stable compounds immobilized in the slag [33,34]. Although effective in immobilizing chlorides, this pyrometallurgical route is energy-intensive and may release corrosive HCl gas, causing equipment degradation and requiring expensive off-gas treatment. Consequently, hydrometallurgical washing remains the preferred industrial practice for dechlorination, while pyrometallurgical methods are reserved for specific applications where downstream integration with thermal processes is feasible.

## 3. Resource Utilization of PAD

In China, most companies focus their resource utilization efforts on PAD. The study of aluminum recovery from PAD was relatively early, and the recovery of metallic aluminum from PAD has high economic value, with the rates of metallic aluminum ranging from 50% to 80%, and the application was relatively mature [9]. The technologies for extracting metal aluminum from PAD mainly include thermal treatment, cold treatment and the combination of both. Although thermal and cold treatment technologies have reached a relatively mature stage, there remains the potential for improving metallic aluminum recovery. The follow-up research can break through from two dimensions: one is to systematically explore and precisely optimize process parameters such as temperature and treatment time, and tap potential effectiveness of existing technology; the other is to focus on the research and development of new separation technology, and further improve the recovery rate of metallic aluminum through technological innovation. From an international industrial perspective, the success of PAD processing is not only measured by the recovery rate but also by the net melt loss and the chemistry of the recovered metal. Net melt loss, which accounts for dross generation rate and recovery in secondary processing, is considered the best indicator of a cast shop’s ability to avoid oxidizing the metal [36]. A major concern for dross processors and their customers is metal contamination. Peterson [36] identified three primary sources for this contamination: (1) in the melting furnace; (2) in the post-melting furnace; and (3) in the rotary furnace during processing. Roth [37] further emphasizes that the historical trend has been a move towards maximizing in-house recovery, with technologies like hot dross pressing and advanced mechanical systems gaining prominence for their ability to produce high-grade metallic concentrates suitable for direct in-house melting, thereby keeping the value of the recovered aluminum within the company.

### 3.1. Thermal Treatment

The core process of the thermal technology is to heat treat PAD by a high-temperature heat source. In a high-temperature environment, the metallic aluminum in aluminum dross is gradually heated up to the melting point and transforms into a molten state; due to its density difference between liquid metallic aluminum and solid impurities, the molten aluminum naturally sinks and gathers at the bottom of the container under gravity; by using a drainage device at the bottom to drain the molten aluminum, effective separation of metallic aluminum and impurities can be achieved, thus completing the recovery and purification of metallic aluminum [38,39]. Electrolytic and remelted aluminum production enterprises generally choose thermal treatment technology to extract aluminum. Thermal treatment of PAD to extract metallic aluminum included the method of fried ash and the method of rotary furnace. The thermal treatment technology is simple to operate and can be used for industrial production. However, the recovery rate of metallic aluminum is low, and the process generates pollutants. Therefore, further optimization is needed to improve recovery and reduce pollutant generation.

**Figure 5:**
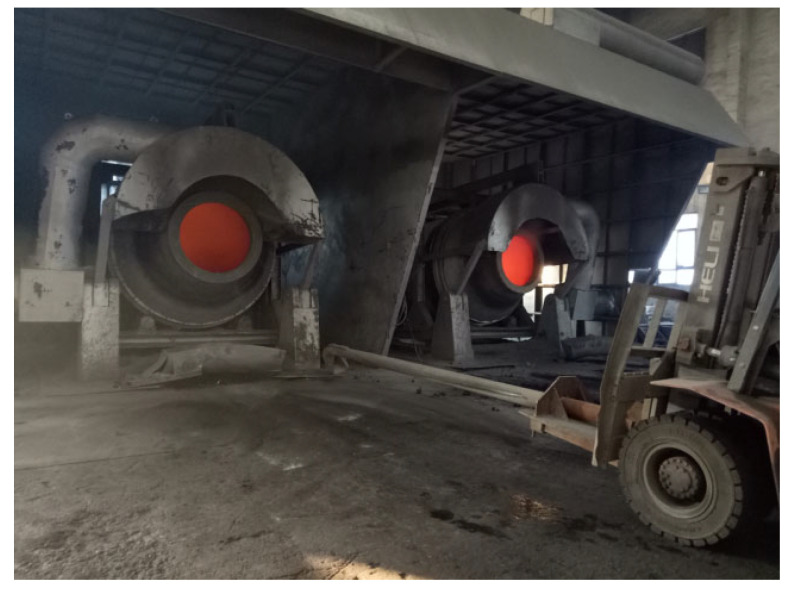
One disposal site of aluminum dross by the fried ash method in China.

The process of the fried ash method is as follows: Pour the hot aluminum dross into an iron pan placed obliquely, and use the heat of aluminum dross to continuously stir it through a mechanical stirring device. By separating the molten aluminum at the bottom of the iron pan, the efficient recovery of metallic aluminum is realized [40]. This method fully utilizes the residual heat of aluminum dross, reducing additional energy consumption, and, at the same time, relies on the wetting of aluminum and the action of gravity sedimentation to achieve the effective separation of aluminum from other impurities and achieve the purpose of recycling metallic aluminum. A typical disposal site of aluminum dross by the fried ash method in China is illustrated in Figure 5.

**Figure 6:**
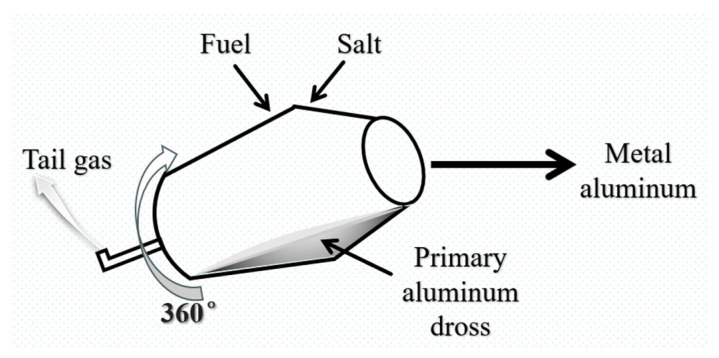
Recovery of aluminum metal by rotary kiln method.

The rotary kiln method and the fried ash method share similarities in their treatment principles, both of which are based on high-temperature treatment to achieve the separation of metallic aluminum from impurities. However, due to the distinct equipment structures employed in the two methods, they exhibit significant differences in operation, treatment efficiency, and application scenarios. PAD contains inherent latent heat. During high-temperature rotary kiln treatment, the hot aluminum dross moves continuously in the rotary kiln and is repeatedly mixed, causing the metallic aluminum to melt completely. The process of recycling metallic aluminum by the rotary kiln method is shown in Figure 6. Due to the poor wettability of aluminum melt and other components in aluminum dross, as well as the large difference in density between them, the melt continues to gather at the bottom of the rotary kiln due to its own gravity and differences in fluidity, and then it flows out continuously, so as to realize the extraction of aluminum. Shao et al. [41] found that the recovery rate of aluminum was about 45% when the reverberatory furnace temperature was between 1000 and 1100 °C, and about 60% when the chamber temperature of the tilting reverberatory furnace was between 700 °C and 750 °C.

During the heat treatment process of aluminum dross, it is necessary to add a certain amount of salt-type flux to promote the separation of aluminum dross liquid aluminum. However, the heat treatment process generates new pollutants, which pose greater challenges for subsequent treatment and significantly increase the treatment difficulty [11]. Lei et al. [42] found that mixing SAD with ammonium sulfate and roasting enables the formation of easily leachable aluminum ammonium sulfate, thereby achieving efficient recovery of aluminum resources. Meshram et al. [43] found that adding salt flux in the process of recovering metallic aluminum by pyrometallurgy has multiple functions: the salt flux can effectively inhibit the oxidation of aluminum at high temperature, allowing more aluminum to precipitate in liquid form, and can also remove impurities, purifying and refining the metallic aluminum. The salt flux can improve the fluidity of the aluminum liquid, reduce the melting point of the melt, and make the metallic aluminum melt at a relatively low temperatures, thereby reducing energy consumption, but it will increase the amount of slag and production costs.

The fried ash method and the rotary furnace method offer significant operational convenience, with a relatively simple process flow that is easy to master and implement. However, these methods have obvious shortcomings in metallic aluminum recovery, and the recovery rate of aluminum is generally low, making it difficult to achieve efficient resource utilization. Moreover, due to process characteristics, the recovery site is prone to dust and high temperatures, exposing operators to harsh working conditions for extended periods and posing occupational safety risks.

### 3.2. Cold Treatment

Cold treatment methods include the mechanical sieving method and electrical selection method. The cold treatment process is simple to operate and low in energy consumption, suitable for small-scale processing, but it has a narrow range of applications and can only handle cold aluminum dross, plus it requires further optimization to improve the recovery rate.

The mechanical sieving method is an efficient and easy-to-operate method for the recovery of aluminum dross. After mechanical crushing and fine milling, aluminum particles and ash are efficiently separated by size using vibrating screens or air flow separation. This method improves the aluminum recovery rate and is suitable for enterprises that process PAD from various sources [40]. The electrostatic separation method is based on the conductivity of aluminum, and it can achieve efficient separation by utilizing the significant difference in conductivity between metallic aluminum and impurity components. Under the action of a high-voltage electric field, the metallic aluminum with good conductivity migrates directionally, while the impurity components are not affected, thereby achieving the purification of metallic aluminum [4]. Compared with other aluminum dross treatment methods, the recovery rate of the electrostatic separation method is relatively low, and it has limitations in application scenarios. It is mainly suitable for the treatment of cold aluminum dross. The technology and equipment are simple and low in energy consumption. Cao et al. [44] used jet airflow dispersion combined with high-voltage corona separation to recover metallic aluminum. After the completion of the three-step electrostatic selection, the metallic aluminum content increased from 8% to 31%.

### 3.3. Cold and Thermal Treatment

**Figure 7:**
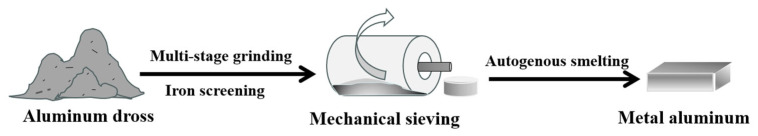
Schematic diagram of efficient recovery of aluminum metal by heat treatment.

Cold–thermal treatment is a metallic aluminum recovery method that combined the thermal treatment method with the cold treatment method, combining the advantages of both thermal and cold treatment. The cold–thermal treatment technology can effectively treat cold aluminum dross that has been stored for a period of time, shows good applicability to cold aluminum dross with different storage periods, and can achieve a high aluminum recovery rate [40]; however, the process is complex and the equipment requirements are high. Aluminum recovery rates can exceed 80% via mechanical sieving and self-heating smelting, and the metallic aluminum extracted by this technology has a low carbon footprint at only 5% of the carbon footprint of the aluminum production [45]. The flow chart of the cold–thermal treatment for efficient metallic aluminum recovery is shown in Figure 7.

### 3.4. Techno-Economic Analysis of PAD Recovery

For PAD, the primary recoverable substance is metallic Al. Processing 1000 kg of PAD can theoretically produce up to 150–750 kg of metallic aluminum. After aluminum extraction, the remaining residue is essentially converted into SAD. Table 6 summarizes the main technologies for recovering metallic Al from PAD, along with their estimated costs, secondary wastes, and the current market price of the recovered product.

The main goal of the resource utilization of PAD is to extract the metallic aluminum in the PAD. Whether it is thermal method treatment or cold method treatment, or a combination of thermal and cold treatment methods, it is necessary to meet the maximum extraction of metallic aluminum, while also prioritizing environmental protection during the extraction process. This is because, after the metallic aluminum is extracted, the remaining PAD residue can be directly classified as SAD and then mixed with other SAD for further utilization. Of course, the research on economical, environmentally friendly, and efficient metallic aluminum separation technology is an important link in current PAD resource utilization.

## 4. Resource Utilization of SAD

**Figure 8:**
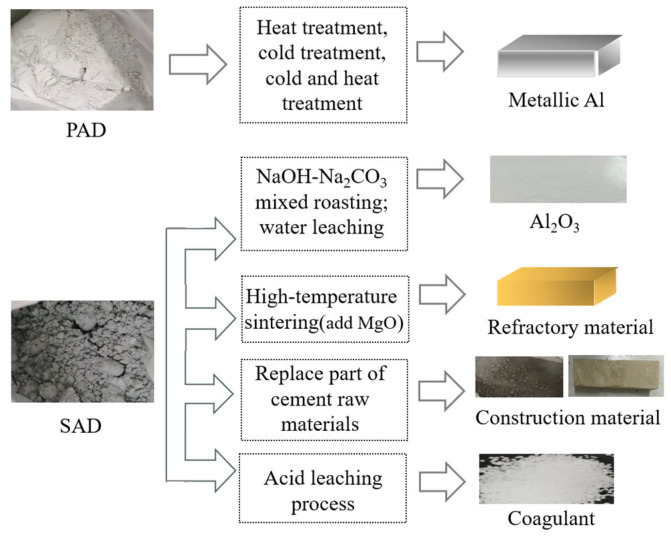
Resource utilization of aluminum dross.

This section focuses on the diversified resource utilization approaches of SAD. To comprehensively summarize various resource valorization routes, Figure 8 illustrates the mainstream technological pathways for resource recovery of both PAD and SAD.

### 4.1. Refractory Material

Refractories typically require a high melting point and good thermal stability. SAD, with its high alumina content, is therefore an important raw material for refractory manufacturing. The application prospects of SAD in the refractory field are broad, offering high resource utilization and excellent refractory properties. However, the process is complex and the equipment requirements are high, necessitating further optimization to improve material performance.

Magnesia–alumina spinel (MgAl_2_O_4_) is a kind of refractory material with excellent properties, such as high melting point, low coefficient of expansion, strong corrosion resistance, good chemical stability, and good heat shock resistance. Li et al. [46] prepared high alumina refractories with MgAl_2_O_4_ as the main phase and CaAl_2_O_4_ as the auxiliary phase by using aluminum dross. Shi et al. [47] found that increasing the magnesium oxide addition significantly improved the bending strength and impact toughness of the material due to improved grain size uniformity and the elimination of microcracks. At a sintering temperature of 1600 °C, they prepared magnesia and magnesia–alumina spinel refractories with an apparent porosity of 18.2% and a volume density of 2.92 g/cm^3^.

Wongsawan et al. [48] utilized the alumina in SAD and magnesia to react at 1600 °C to generate magnesia–alumina spinel, and the iron oxides in the mill scale also participated in the reaction to generate iron aluminate spinel (FeAl_2_O_4_) and magnesia–iron–alumina spinel (MgFeAlO_4_), the presence of these spinel phases significantly improved the stability and mechanical strength of the refractory material, and the cold-pressed strength of the samples ranged from 76.79 MPa to 95.67 MPa. Kongkajun et al. [49] successfully synthesized iron aluminate spinel by mixing SAD, mill scale, and graphite and carrying out the combustion reaction in an air atmosphere at 1550 °C, and the properties of the reaction products were comparable to those of commercial iron aluminate spinel refractory materials.

Zhang et al. [13] utilized SAD, calcium oxide, and magnesium oxide, sintering at a high temperature of 1500 °C for 3 h, to prepare a calcium–aluminum orthoclase/magnesium–aluminum spinel composite with an apparent porosity of 33.87% and a compressive strength of 40.18 MPa. Ai et al. [50] used SAD and industrial heavy-burned magnesia sand to prepare magnesia–alumina spinel refractory materials with a compressive strength of 42.81 MPa. Zhou et al. [51] used a mass ratio of 3:3:4 for SAD, red mud, and silicate tailings, sintered at 1120 °C for 2 h, to prepare ceramic sintered bricks. This method produced sintered bricks with excellent properties, achieving a compressive strength of 83.04 MPa, and demonstrated the synergistic utilization of SAD with other waste residues, providing new ideas for the preparation of refractory materials. Adeosun et al. [52] prepared insulation refractory bricks by mixing aluminum dross into plastic clay and kaolin, which withstood temperatures up to 1200 °C. Lightweight refractory materials can also be prepared using SAD and fly ash, with the addition of water reducers and binders. The high alumina content of aluminum dross and the synergistic effect of the silica–alumina ratio in fly ash can effectively improve the strength and heat resistance of refractory materials.

Hou et al. [53] prepared magnesia–alumina spinel refractories by the acid leaching of SAD, mild desilication, and compounding with light-burned magnesia to form a magnesia-rich sintering system. When the addition of light magnesium oxide was 20%, the sintering temperature was 1600 °C, and the sintering time was 3 h, the magnesia–alumina spinel with a volume density of 2.76 g/cm^3^ and an apparent porosity of 21.85% was prepared. Huang et al. [54] used SAD as the raw material and prepared high-performance magnesia–alumina spinel–corundum composite refractories after alkali leaching, impurity removal with dilute acid, and sintering.

Compared with refractory bricks, ceramics offer wider economic benefits. Liu et al. [55] added different amounts of nickel ferrite slag, and it was found that with the increase in the addition of nickel ferrite slag, the content of violet and the microstructure densification of mullite were promoted, and the porosity of violet–mullite ceramics decreased from 41.7% to 34.4%, and the compressive strength of violet–mullite ceramics reached 52.8 MPa when the addition of nickel ferrite slag was 20%. Zhu et al. [56] synthesized β-Sialon ceramics by nitriding the reduction reaction with SAD as the raw material, and they found that with the increase in Si/Al, the aspect ratio of β-Sialon grains increased rapidly, and the β-Sialon grains eventually became fibrous. Zawrah et al. [57] prepared mullite dense ceramics with SAD and silica fume: when the content of silica in the material increased, the porosity and bulk density decreased; however, when increasing the temperature from 1300 °C to 1500 °C, they showed the opposite trend; and, when the mullite content in the material increased, the micro-hardness of the specimen with high mullite content was 10.2 GPa and the compressive strength was 16.9 MPa.

To date, research has proved that a variety of high-performance refractories such as magnesia–alumina spinel, firebrick and ceramics can be successfully prepared with SAD as the main raw material. These materials not only exhibit excellent high-temperature resistance but also their mechanical strengths that meet industrial application requirements. The application of aluminum dross in refractory production can significantly reduce production costs, but its impurity content is high. It is easy to form low melting compounds or glass phase in actual production, which leads to the deterioration of material properties; the current technology focuses on high-temperature sintering, and future research should shift focus toward developing novel sintering aid systems. On the basis of maintaining the stability of material properties, the sintering temperature can be effectively reduced, thereby reducing energy consumption. In addition, it is also necessary to develop more efficient pretreatment processes to address the issues of the existing pretreatment process that had a complex technical route and was difficult to industrialize.

### 4.2. Building Materials

Many studies have shown that aluminum dross has the potential to be used in preparing high-performance construction materials, which can be used to prepare a variety of materials, such as bricks, cement, and porous ceramics, and can improve the performance of materials, realize the resource utilization of waste, and reduce environmental pollution. In the future, the process should be optimized, and the research on long-term performance should be strengthened to promote the large-scale application of aluminum dross in construction materials.

Ren et al. [58] utilized the ultra-high pressure contact molding technology, with SAD and calcium carbide slag as raw materials, to prepare high-performance bricks. Through experiments, it was concluded that the compressive strength of the prepared building materials was 60 MPa, and flexural strength was 1.3 MPa when SAD was used as raw material and naturally cured under 300 MPa pressure for 3d. Guo et al. [59] prepared non-clay bricks using SAD via waste glass as raw materials and high-temperature sintering. The waste glass promoted the formation of high-density ceramic phases and also enhanced the performance of the brick body. The active components in aluminum dross can react with calcium ions in cement to generate calcium silicate hydrate gel, which can improve the strength and durability of concrete. Aluminum dross can effectively improve the workability and mechanical properties of cement as a cement admixture. The aluminum oxide and silicate components in aluminum dross can replace part of the cement raw materials, reduce the cost of cement production, and improve the early strength and durability of cement [60]. Ni et al. [61] found that the optimum mix ratio for preparing unburned bricks from SAD was 68.3% SAD, 11.4% cement, 6.4% slaked lime, 4.2% gypsum, and 9.7% engineering sand. The compressive strength, frost resistance, and water absorption of the resulting unburned bricks all reached the MU20 performance level for unburned building bricks. The results of this study provide a theoretical basis for the application of aluminum dross in preparing unburned bricks, which can effectively reduce aluminum dross stockpiles and environmental pollution.

**Figure 9:**
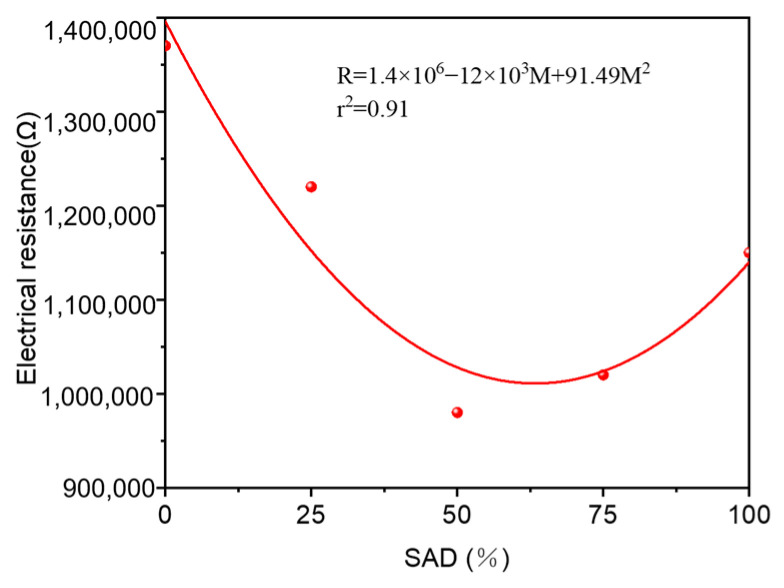
The electrical resistance of UHPC with different dosages of SAD [62].

Sun et al. [62] investigated the influence of SAD and waste fly ash on the performance of ultra-high-performance concrete (UHPC), and found that the UHPC exhibited the best performance when the mass ratio of SAD to waste fly ash was 1, the resistivity reached its lowest value, the slump reached 234.6 mm, the minimum plastic viscosity was 10.7 Pa∙s, and the minimum yield shear stress was 2.3 Pa. At the same time, SAD delayed the initial setting time of concrete, and the workability and fluidity were significantly improved, and the overall performance also enhanced. Figure 9 shows the electrical resistance of UHPC as a function of SAD dosage.

**Figure 10:**
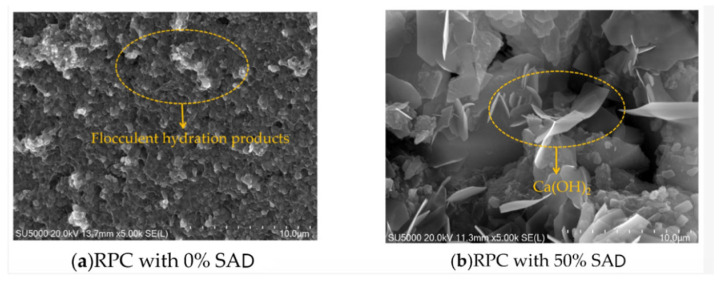
SEM of RPC at different SAD additions [61].

Ba et al. [63] investigated the influence of SAD sintered grinding powder on the performance of sulphoaluminate cement-based grouting materials and found that the comprehensive performance was optimal at 15% SAD sintered grinding powder content, which could promote cement hydration and stabilize heavy metals, but the long-term compressive strength would decrease with the increase in dosage. Xu et al. [64] utilized aluminum dross generated during the production of aluminum sulfate coagulant production to prepare composite cement, with aluminum dross content between 8% and 10%. Xu et al. [65] found that the addition of SAD can improve the electrical conductivity of freshly mixed reactive powder concrete (RPC), and the incorporation of SAD can significantly change the microstructure of the specimen, reduce the flocculation of hydration products, and produce large amounts of hexagonal Ca(OH)_2_. In addition, as shown in Figure 10, the increased hydration products improve the compaction degree of RPC after 28d curing, thereby improving the mechanical strength.

Liu et al. [66] synthesized a porous metal ceramic composite with a compressive strength of 17.77 MPa by a thermal treatment process using SAD and red mud as raw materials. And porous ceramic materials with a compressive strength of 12.44 MPa were also prepared with SAD and calcium oxide as the main raw materials. Pan et al. [67] investigated the influence of SAD replacing cement in concrete and found that SAD should not be used in C60 high-strength concrete, but when SAD replacing cement does not exceed 10%, it does not significantly affect the compressive strength of C30 concrete and can reduce construction costs.

A particularly promising application for the non-metallic residue from SAD is its use as a raw material in the production of Portland cement clinker. The major components of clinker feed are lime, silica, alumina, and iron oxide, making the alumina-rich SAD residue a potential substitute for natural bauxite. Tsakiridis et al. [68] conducted a series of studies on this subject, in which a black dross leached residue—obtained after hydrothermal treatment to remove salts and nitrides—was used to replace 2 and 4 wt% of the raw meal. The results showed that the produced cements met the required mechanical properties and had comparable hydration behavior to the reference sample. In a later study, salt slag washed residue was used to fully replace bauxite (4.5 wt%) in the raw meal, successfully producing a clinker with a C3A content of 11.2% and a final cement that satisfied the requirements for the 52.5 strength class [69]. These studies demonstrate that, after proper pretreatment to remove harmful components like halides and nitrides, SAD or salt slag residues can be effectively and safely utilized as a valuable resource in cement manufacturing, offering a sustainable alternative to landfilling.

In the field of building materials, significant progress has been made in the application research of SAD. However, it is worth noting that potential heavy metals in aluminum dross may leach out during the long-term service of building materials, posing risks to the environment and human health. Therefore, a systematic assessment should be conducted before the large-scale application of aluminum dross in building materials, and its long-term performance under different environmental conditions should be investigated in depth, and targeted technologies should be developed. Such assessments, however, are currently hampered by the lack of nationally unified standards for leaching test protocols and acceptable threshold values, which makes cross-study comparisons and regulatory decisions difficult. Through technological innovation and process optimization, potential safety hazards should be effectively eliminated, and technical support should be provided for the large-scale application of aluminum dross in building materials.

**Figure 11:**
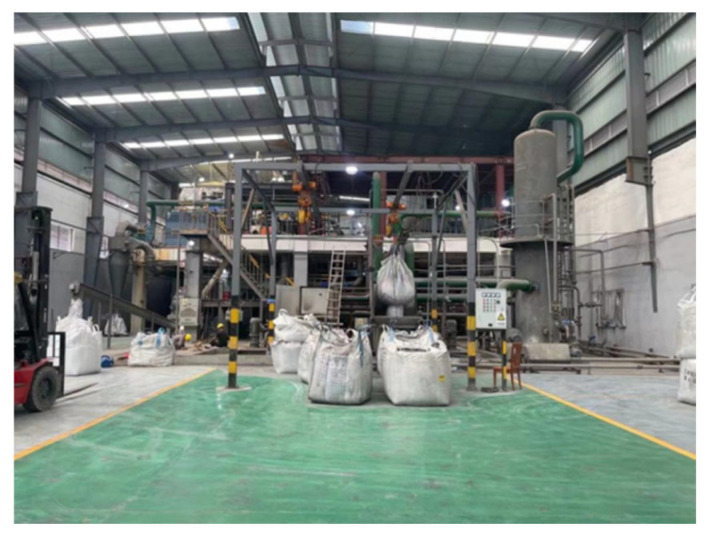
Aluminum dross harmless disposal production line built by a new material company in Zhejiang.

A new material technology company in Zhejiang Province has built the first large-scale industrial process and equipment with completely independent intellectual property rights in China, as shown in Figure 11. Through the breakthrough of key technologies, it has realized the efficient resource conversion of aluminum dross, which can not only recover the aluminum element in it, but also deamination and fix nitrogen, turning the ash residue into new building bricks. The whole process has zero wastewater and waste discharge, and the comprehensive utilization rate of aluminum dross reached 100%, which achieved the purpose of extending the green industry chain.

### 4.3. Coagulant

The applicable scope of coagulants is wide, not only can it be used for the purification treatment of drinking water but also for comprehensive treatment of domestic sewage, as well as also playing an important role in industrial wastewater treatment [70]. Polyaluminum chloride (PAC) is coagulant, and calcium aluminate (CA) with high activity is an important raw material for aluminum-based coagulants, which can be used to prepare high-quality polyaluminum chloride. Aluminum dross is a major by-product of the electrolytic aluminum industry, and, in recent years, its application in the production of coagulants has gradually attracted attention. Aluminum dross contains large amounts of aluminum and alumina, and these components can be used as materials for the production of water treatment agents. To date, PAC has been successfully prepared using alumina in SAD, and the reactions are shown in Equations (6)–(8) [71]:

Al_2_O_3_ + HCl → Al_2_(OH)_n_Cl_6−n_, (6)

CaO + HCl → CaCl_2_ + 2H_2_O, (7)

mAl_2_(OH)_n_Cl_6−n_ → [Al_2_(OH)_n_Cl_6−n_]_m_, (8)

Aluminum sulfate can undergo hydrolysis and polymerization in water, which can effectively remove color, bacteria, impurities, and eliminate odors from water. Therefore, it is a commonly used coagulant in the water treatment industry. Yang et al. [72] studied the production of aluminum sulfate from SAD, investigated the effects of various factors on production efficiency, adjusted the leaching process parameters, reduced the leaching temperature, and achieved an aluminum leaching rate as high as 90.1%, thus obtaining high-quality aluminum sulfate products.

Zhong et al. [73] investigated the process of producing liquid polyaluminum chloride from SAD. By detecting PAC samples produced by different enterprises, it was found that the residual concentration of fluoride and heavy metals (such as Mn, Zn, Cu, and Ni) in some samples met the comprehensive discharge standard for sewage and complied with the environmental protection requirements for resourcefulness and harmlessness. The leaching concentration of the above five pollutants are directly compared with the standard limits, as shown in Table 7.

Zhu et al. [74] investigated the preparation of an iron-based catalyst (FAS) from SAD residue for the photocatalytic degradation of tetracycline in water, which achieved a removal rate of 92.2% for tetracycline under visible light irradiation and maintained high catalytic activity after multiple cycles of use. Aluminum dross can also be used in wetland systems for the removal of nutrients (such as phosphate and ammonia) from water. Mittal et al. [75] investigated the encapsulation of aluminum dross in calcium alginate beads for the removal of phosphate and ammonia in vertical flow constructed wetland (VF-CW) and floating constructed wetland (FCW) systems, and the removal rates of phosphate and ammonia by aluminum dross beads were 85% and 93.44%, respectively, in the VF-CW system. In the FCW system, the removal rates of phosphorus and ammonia were 79.18% and 65.45%, respectively. Additionally, the use of aluminum dross beads can also inhibit the growth of algae and reduce the risk of eutrophication.

By making rational use of the aluminum and alumina in the SAD, SAD can be converted into a water treatment agent, which can not only realize the resource utilization of aluminum dross but also effectively remove pollutants from the water, reducing environmental pollution. However, pollutant residues in SAD and optimization of the treatment process remain key areas for future research. With further research and technological improvement, strengthening the monitoring of heavy metal residues in the final product and establishing strict quality control, the application of SAD in coagulants will be more extensive and efficient.

### 4.4. Extract Alumina

The extraction process of alumina from SAD mainly includes the alkali sintering method, alkali leaching method, acid leaching method and alkali–acid combined method. However, these technologies face challenges such as low extraction efficiency of alumina, high energy consumption and material consumption, large amounts of waste residue and complex process flow, which limit their industrial application. The alkali sintering process shows significant technological advantages in the treatment of aluminum dross: compared with the alkali leaching method, the alkali sintering process uses less alkali and has a higher extraction efficiency in the treatment of aluminum dross, and therefore does not need to use acid-resistant materials for equipment, which greatly reduces maintenance costs. However, the acid leaching method is not as effective as the alkali sintering method in terms of alumina purity. In terms of the complexity of the process flow, the alkali–acid combined method is not as concise and efficient as the alkali sintering method. The alkaline sintering method was more feasible for industrial promotion and application [76].

Based on the current development trend of technology, the key points of future research can be focused on the following points: (1) develop new composite additives to reduce the temperature required for the alkali sintering process through synergistic effect, thereby achieving energy savings and reduced consumption; (2) research selective leaching technology that utilize differences in chemical properties to accurately separate impurities and improve the purity of alumina products from alkali and acid leaching processes; (3) strengthen comprehensive resource utilization research on waste residues from the extraction process, achieving high-value-added utilization of solid waste through technological innovation and promoting efficient resource conversion and circular utilization throughout the process.

#### 4.4.1. Alkali Sintering Method

The alkali sintering process requires high energy consumption and causes severe corrosion of equipment. The impurity elements (such as Fe and Si.) in aluminum dross affect the extraction purity of alumina and increase the difficulty of subsequent treatment. Two activator systems are mainly used in the alkali sintering process: (1) NaOH sintering activation of aluminum dross has a low reaction temperature, fast reaction rate, and generates small amounts of waste residue, but strong alkali corrosion easily leads to poor durability of equipment and high costs. (2) Na_2_CO_3_ sintering activation of aluminum dross had outstanding performance in environmental protection, economy and equipment friendliness, effectively reducing treatment costs and equipment corrosion. However, it requires a higher sintering activation temperature, which has become a constraint to its application.

Ren et al. [76] investigated the process of extracting Al_2_O_3_ from SAD by roasting with NaOH-Na_2_CO_3_ mixed auxiliary agents. The results showed that the leaching rate of Al_2_O_3_ reached 92.29% when roasting at 800 °C for 60 min and leaching at 80 °C for 1 min with a liquid–solid ratio of 15. Tripathy et al. [77] used a single dilute alkali base leaching route for the extraction of alumina roasting activation of aluminum dross. It was found that the mixing of NaOH and Na_2_CO_3_ could produce a synergistic effect, and the mixed auxiliary agent reduces the eutectic point of the roasting system. At room temperature, the activation reaction is mainly promoted by NaOH. Conversely, under high-temperature conditions, Na_2_CO_3_ plays a key activation role. The simultaneous use of the two auxiliary agents promotes the full contact of SAD with the auxiliary agents, so that the leaching rate of Al_2_O_3_ continues to increase. Wu et al. [78] also used the alkali-roasting method to investigate the influence of sintering temperature, alkali-to-alumina ratio, sintering time and other factors on the leaching rate of alumina from SAD. The results showed that the leaching rate of alumina reached 94.23% when the alkali-to-alumina ratio was 1.1 and sintering time was 40 min at 1423 K.

#### 4.4.2. Alkali Leaching Method

The alkali leaching method mainly utilizes the amphoteric characteristics of alumina, and dissolves the alumina in aluminum dross using an alkaline solution (such as sodium hydroxide). The leaching rate is affected by factors such as the concentration of the alkali liquor, leaching temperature, and liquid–solid ratio. Tsakiridis et al. [79] investigated the influence of alkali concentration and temperature on the leaching rate of alumina from SAD, and found that the leaching rate of alumina reached 98.6% in a 250 g/L alkaline solution, with a leaching temperature of 250 °C and a leaching time of 100 min. Li et al. [80] studied the effect of temperature on the leaching rate of alumina, and found that the leaching rate of alumina reached 98.6% at 250 °C. The advantages of the alkali leaching method are that the extracted aluminum product has fewer impurities, and other metal oxides in aluminum dross do not react with the alkali liquor, so the alkali liquor can be recycled. However, the disadvantage of the alkali leaching method is that the leaching temperature was relatively high (usually above 200 °C), resulting in high energy consumption [79,80].

#### 4.4.3. Acid Leaching Method

The common acids used in the acid leaching method are hydrochloric acid and sulfuric acid, which react with aluminum dross to release Al^3+^, from which alumina is subsequently generated. Li et al. [81] conducted an SAD leaching experiment, using 1–5 mol/L hydrochloric acid, with a liquid–solid ratio of 20, and leaching for 120 min at 75 °C; the leaching rate of alumina reached a maximum of 78.56%. Mahinroosta et al. [82] also conducted SAD leaching experiments with different concentrations of hydrochloric acid, and the leaching rate of alumina showed a first increasing and then decreasing trend. The same trend was found by Dash et al. when they used sulfuric acid [83]. The leaching rate of alumina showed a trend of first increasing and then decreasing, which can be attributed to three factors: (1) when the acid concentration exceeds a certain threshold, it will have an inhibitory effect on the ionization process of H^+^, which will reduce the leaching efficiency; (2) excessively high acid concentration promotes the competition of metal cations with Al^3+^ for Cl^-^ coordination; and (3) excessively high acid concentration accelerates the dissolution of Al^3+^ will inhibit the diffusion of H^+^. Liu et al. [84] adopted the process of acid leaching–aluminum ammonium carbonate crystallization to achieve an efficient recovery of alumina from aluminum dross, achieving an alumina leaching rate of 91.5%. Acid leaching has the advantages of a simple process and less generation of residue, but there was a phenomenon of co-dissolution of non-aluminum impurities such as Fe and Si in the acid leaching process, which leads to low purity of the alumina product.

#### 4.4.4. Alkali–Acid Combined Method

The alkali–acid combined method combines the advantages of acid leaching and alkali leaching, first dissolving the alumina in aluminum dross through acid leaching and then further purifying it by alkali leaching. Mahinroosta et al. [82] achieved an alumina leaching rate of 83% and a purity of 97.61% after calcination at 700 °C using this approach, while An et al. [85] recovered 78.56% of alumina with a purity as high as 99.2%. Although this method offers high product purity and yield, its drawbacks include a cumbersome process, high acid/alkali consumption, and limited scalability. Tsakiridis et al. [79] also developed a comprehensive hydrothermal process for treating black dross, which includes a final step for alumina recovery. The process consists of crushing and grinding, sieving to recover metallic Al, water leaching at high temperature to dissolve and recover salt flux, and, finally, alkaline pressure leaching to recover the remaining aluminum. At optimized conditions (260 g/L NaOH, 240 °C, 100 min), about 57.5% of the aluminum was leached from the washed residue. This approach not only recovers valuable salts and aluminum but also produces a leached residue that is free of chlorides and nitrides, making it suitable for further applications such as the cement production mentioned above. This integrated process exemplifies a ‘zero-waste’ strategy, where all components of the SAD are valorized into valuable products.

Resource recovery from SAD is the final step in the overall resource utilization of aluminum dross. Its resource utilization technology level directly affects the work of resource utilization of aluminum dross in China. The resource utilization of SAD should first aim at its resource attribute, which is to analyze its physical and chemical properties, to open up a wide range of utilization channels, and to try in many fields, such as refractory materials, building materials, water treatment agents and the extraction of alumina. Although China’s industrialization of SAD resource utilization is still in the exploratory stage, it is necessary to pay more attention to environmental pollution control during treatment, while optimizing processes and reducing costs, in order to promote the sustainable development of China’s aluminum industry through more rational processes. However, the absence of unified national standards for product quality and environmental safety remains a major barrier to large-scale application, leaving both producers and end-users without clear benchmarks for performance evaluation and risk control.

### 4.5. Techno-Economic Analysis and Policy Recommendations

To bridge the gap between laboratory research and industrial application, a preliminary techno-economic assessment is essential. Based on a typical composition of SAD (Table 1) and assuming ideal 100% recovery of its valuable components, the relevant techno-economic parameters are summarized in Table 8.

## 5. Conclusions

(1) The resource utilization of aluminum dross is an important direction for promoting the detoxification of hazardous solid waste and the development of a circular economy in China’s aluminum industry. Primary aluminum dross (PAD) can achieve high metallic aluminum recovery through combined cold–thermal processes, while secondary aluminum dross (SAD) has been successfully used to prepare refractory materials, building materials, coagulants, and alumina. These routes not only reduce environmental pollution but also generate considerable economic benefits compared with traditional landfilling or stockpiling.
(2) The main technical challenges originate from the complex and variable composition of aluminum dross, which leads to inconsistent product performance. Existing pretreatment processes (hydro- or pyro-methods) suffer from either low denitrification efficiency or high energy consumption. Moreover, there is a lack of unified national standards for detoxification and resource-derived products, as well as insufficient long-term environmental risk assessment for SAD-based construction materials and coagulants (e.g., heavy metal leaching behavior under real service conditions).
(3) To systematically advance this field, future efforts should focus on: establishing long-term leaching monitoring and life-cycle assessment models for aluminum dross-based products; accelerating the formulation of classification standards, usage specifications, and environmental release thresholds for PAD and SAD; and developing a “cascade utilization” model—first extracting metallic aluminum from PAD, then converting the remaining SAD into refractories or building materials, and finally recovering alumina from the residual slag. Only through coordinated innovation in pretreatment technology, standard refinement, and risk management can high-value, large-scale, and environmentally safe utilization of aluminum dross be achieved.

## Figures and Tables

**Table 1:** Typical compositional ranges and formation sources of PAD and SAD (wt%) [7,11,12].

Component	PAD	SAD	Main Source
Metallic Al	15–75	2–15	Entrapped molten metal during skimming
Al_2_O_3_	25–30	30–80	Surface oxidation; accumulates as other components are removed
AlN	<5	10–30	Metal aluminum reacts with N_2_ in the air in a molten state to generate
Soluble salts	15–30	5–15	Flux added to prevent oxidation; partially washed out during PAD processing
Fluorides	<1	1–6	The flux used in the electrolytic cell is mixed with aluminum dross
Chlorides	0.1–4	0.2–4.3	From salt flux addition
Magnesium compounds	3–15	8–23	Oxidation of Mg in Mg-bearing Al alloys

**Table 2:** Chemical composition of SAD from different sources (wt%).

Component	Yunnan SAD [12]	Shandong SAD	Zhejiang SAD
Al_2_O_3_	34.90	78.91	72.74
MgO	8.62	11.22	13.26
SiO_2_	1.93	1.53	2.07
Na_2_O	4.92	0.50	2.30
K_2_O	0.12	2.48	1.95
CaO	2.56	0.29	0.45
Fe_2_O_3_	16.58	0.20	0.42
F	5.38	3.74	1.87
Cl	—	0.39	3.58
AlN	16.8	—	—
Others	8.19	0.74	1.36

Note: “—” indicates that the corresponding component was not detected in the sample.

**Table 3:** Removal of nitrogen from aluminum dross.

Methods	L/S	T/°C	t/min	Removal Rate/%	Ref.
NaOH leaching (1.6 mol·L^−1^)	12	80	30	40.0	[18]
NaOH leaching (4%)	6	95	180	96.2	[19]
Water leaching 10% CaO + 0.75% glucose	10	90	120	95.4	[20]
Water leaching	1.5	80	300	84.8	[21]
Water leaching	10	90	480	87.6	[22]
Water leaching	10	90	720	95.3	[23]
Roasting with Na_3_AlF_6_ (17.7%)		750	194	94.7	[24]
Roasting with CaO (40%)		900	300	85.3	[25]
Roasting with O_2_		800	120	51.5	[26]
Roasting with O_2_		1200	120	89.1	[26]
Roasting with O_2_		1200	240	93.8	[26]

Note: All % in the first column of the table indicate mass fraction.

**Table 4:** Removal of fluorine from aluminum dross.

Methods	L/S	T/°C	t/min	Removal Rate/%	Ref.
NaOH leaching (4%)	6	95	180	69.2	[19]
NaOH leaching (4%)	4	60	480	87.7	[31]
NaOH leaching (1.5 mol/L)	10	90	30	65.9	[32]
Water leaching	10	90	480	93.4	[22]
Water leaching	30	90	240	13.0	[32]
Roasting with CaCl_2_ (3%)		1300	240		[24]
Roasting with CaO + NaOH		1000	120	99.7	[33]
CaCl_2_ leaching(24%)	8	28	60	99.8	[33]

Note: All % in the first column of the table indicate mass fraction.

**Table 5:** Removal of chlorine from aluminum dross.

Methods	L/S	T/°C	t/min	Removal Rate/%	Ref.
Roasting with Na_2_CO_3_ or CaO		1150	60	95.4	[34]
Roasting with NaOH + CaO		1000	120	99.7	[33]
Water leaching	10	90	480	98.4	[22]
Water leaching	8	80	900	99.7	[33]
NaOH leaching(4%)	6	95	180	95.6	[21]
NaOH leaching(10%)	4	80	20	95.8	[35]

Note: All % in the first column of the table indicate mass fraction.

**Table 6:** Techno-economic parameters for recovering metallic Al from PAD.

Technology	Secondary Waste	Estimated Cost (USD/ton PAD)	Market Price (USD/ton Al)
Thermal treatment	Salt slag, dust, SAD residue	80–120	3400
Cold treatment	Fine dust, SAD residue	60–100	3400
Combined cold–thermal treatment	Minimal salt slag, SAD residue	100–150	3400

**Table 7:** Comparison of leaching concentration of pollutants in polyaluminum with standard limits [73].

Pollutant	F	Mn^2+^	Zn^2+^	Cu^2+^	Ni^2+^
Concentration range (mg/L)	0.20–0.51	0.05–0.32	0.20–0.73	0.24–1.11	0.11–0.80
Surface water standards (mg/L)	1.0	0.1	1.0/0.05	1.0/0.1	0.02
Standards for drinking water (mg/L)	1.0	0.1	1.0	1.0	0.02
Comprehensive sewage discharge (mg/L)	10	2.0	2.0	0.5	1.0

**Table 8:** Techno-economic parameters for recovery of valuable resources and elimination of hazardous components from per ton SAD.

Category	Item	Theoretical Yield (kg)	Technology	Estimated Cost (USD)	Secondary Waste	Market Price (USD)
Resource recovery	Al_2_O_3_	300–800	NaOH-Na_2_CO_3_ mixed roasting; water leaching	120–180	Alkaline residue	400–600
	MgAl_2_O_4_ (Spinel)	500–1000	High-temperature sintering with light MgO	200–300	Solid residue; off-gas	800–1200
Hazard removal	AlN	100–300	Hydro-method; Pyro-method	30–80	Wastewater; off-gas	-
	F	10–60	Hydro-method; Pyro-method	40–90	Wastewater; CaF_2_	-
	Cl	2–43	Hydro-method; Pyro-method	40–60	Wastewater; off-gas	-

Note: “-” indicates that the corresponding market price cannot be estimated.

## Data Availability

No new data were created or analyzed in this study. Data sharing is not applicable to this article.
